# A grid-based database on vascular plant distribution in the Meshchersky National Park, Ryazan Oblast, Russia

**DOI:** 10.3897/BDJ.9.e75892

**Published:** 2021-11-01

**Authors:** Andrey V. Shcherbakov, Marina V. Kazakova, Nadezhda V. Lyubeznova, Anastasia D. Pastushenko, Alexey P. Seregin

**Affiliations:** 1 Lomonosov Moscow State University, Moscow, Russia Lomonosov Moscow State University Moscow Russia; 2 Ryazan State University named for S. A. Esenin, Ryazan, Russia Ryazan State University named for S. A. Esenin Ryazan Russia

**Keywords:** vascular plants, grid mapping, dataset, flora, Ryazan Oblast, Russia, occurrence

## Abstract

**Background:**

Ryazan Oblast, situated in the central part of European Russia, has a long tradition of biodiversity research. Large distributional, ecological and phenological data on various taxonomic groups are available from this territory, mainly in the form of paper publications items, undigitised museum collections and archival sources. The purpose of this dataset is to deliver floristic materials, collected by the authors in the Meshchera Lowlands in the form of GBIF-mediated electronic data, to a wider audience. The dataset covers wild tracheophytes (native species, naturalised aliens and casuals) of the Meshchersky National Park. In 2020, it was used for the production of grid maps in "Flora of the Meschchersky National Park: checklist and atlas".

**New information:**

The dataset contains 14,476 grid records of 817 taxa (806 species and hybrids, ten species aggregates and one genus). Most of the records (82.4%) were made in the field by A.V. Shcherbakov, M.V. Kazakova, N.V. Lyubeznova and A.D. Pastushenko in 2017 and 2018. The dataset includes only one occurrence per species per grid square. Georeferences are based on the WGS84 grid scheme with 55 squares measuring ca. 25 km^2^ (2.5' lat. × 5' long.). Each occurrence is linked to the corresponding grid square centroid; therefore, actual coordinates, habitat details and voucher information are unavailable. As of September 2021, the dataset on the flora of the Meshchersky National Park represents the second largest dataset on the biodiversity of Ryazan Oblast, Russia, published in GBIF.

## Introduction

Grid data are forming some major datasets on vascular plants in the Global Biodiversity Information Facility (GBIF). They are mostly coming from European countries with a long tradition of national biodiversity documentation and mapping. For instance, datasets for France (Bruno and Solène 2016, Hendoux et al. 2019), Germany (Anonymous 2021a, Anonymous 2021b, Anonymous 2021c), Finland (Lampinen and Laiho 2021), The Netherlands (de Vries and Lemmens 2021) and Belgium (Van Landuyt et al. 2012, Van Landuyt and Brosens 2021) are especially noteworthy as for number of records and complexity of initial data. Other datasets, like those from The Netherlands (Hennekens 2018), the United Kingdom (Anonymous 2021d, Anonymous 2021e), Switzerland (Jutzi et al. 2021) and Spain (REDIAM. Red de Información Ambiental de Andalucía 2019), combine grid data with precise georeferences.

In Russia, at least five grid datasets on vascular plants were published earlier in GBIF. They include two datasets with 5' lat. × 10' long. grid squares for Vladimir Oblast (Seregin 2021a, Seregin 2021b) and Samoylovsky District of Saratov Oblast (Pashkina 2019) and two datasets with smaller grid squares 2.5' lat. × 5' long. for the southern tip of Yaroslavl Oblast (Frontova 2019) and the Meshchera National Park (Seregin 2014). A grid dataset for Udomelsky District, Tver Oblast (Abramova and Volkova 2018) is using a stand-alone grid system with 5 × 5 km squares.

The present dataset (Shcherbakov et al. 2021) is a geographical extension of "A grid-based database on vascular plant distribution in the Meshchera National Park, Vladimir Oblast, Russia" (Seregin 2014). Meshchera National Park in Vladimir Oblast and Meshchersky National Park in Ryazan Oblast are twin parks established simultaneously within two subjects of the Russian Federation. Currently, they have a single administration, located in Gus-Khrustalny, Vladimir Oblast.

The study on vascular plants in the Meshchera National Park, Vladimir Oblast was performed mostly in 2002 and 2012 by Dr. A.P. Seregin. Later on, these results were published as two printed atlases (Seregin 2004, Seregin 2013) supported by a GBIF-mediated dataset (Seregin 2014). In 2017-2018, a similar field grid survey was performed by Dr. A.V. Shcherbakov, Prof. M.V. Kazakova, Dr. N.V. Lyubeznova and Dr. A.D. Pastushenko in the Meshchersky National Park, Ryazan Oblast. These efforts resulted in the publication of another printed atlas (Shcherbakov et al. 2020). Data used for the final map production in the latter atlas were transformed into a GBIF-mediated dataset (Shcherbakov et al. 2021) described in this datapaper.

As of 22 September 2021, the Meshchersky National Park plant occurrence dataset created the second largest dataset on the biodiversity of Ryazan Oblast published in GBIF after the Moscow University Herbarium (Seregin 2021c).

## Project description

### Title

Grid mapping of vascular plant distribution in the Meshchersky National Park, Ryazan Oblast, Russia

### Study area description

The Meshchersky National Park was created in Ryazan Oblast by the decree of the Government of the Russian Federation No. 235 dated 09.04.1992. Since 2015, the Park has a joint administration with adjacent Meshchera National Park, Vladimir Oblast. The Park is located in the north-western corner of the Ryazan Oblast within Klepikovsky District (948.24 km^2^) and Ryazansky District (81.9 km^2^). The area of the park is 1,030.14 km^2^, of which 486.06 km^2^ (47.2%) fall on the lands of the State Forest Fund managed by the Park and 544.08 km^2^ (52.8%) belong to other users and are included into the National Park without exemption from economic activities. The local administration of the Park is located in Spas-Klepiki. Small offices are situated in Grishino, Prudki and Shakino (Shcherbakov et al. 2020).

The Meshchersky National Park is located in the middle of the Meshchera Lowlands, also referred to as simply Meshchera (Russian "Мещёра"), a spacious lowland in the middle of European Russia. The Meshchera Lowlands occupies adjacent parts of Moscow Oblast, Vladimir Oblast and Ryazan Oblast. It is named after the Finnic Meshchera people, who used to live in this territory and were later assimilated by the Russians. Meshchera is a plain of roughly triangular shape bounded by the Oka River from the south and east, the Moskva River from the southwest and the Klyazma River from the north. Mean elevations are 80–130 m above sea level (Alexeev et al. 1986).

The climate of the Meshchera Lowlands is humid continental with long, cold and snowy winters and short, warm and rainy summers. Annual average temperature is +4.3°C. The coldest month is January or February with average temperature of −11.6°C. During severe winters, temperatures can go as low as −47°C. Summers are warm, sometimes hot, with average July temperature of +19.8°C and, in extremely hot summers, the temperature can rise up to +40°C (Alexeev et al. 1986, Shcherbakov et al. 2020).

Typical features of the national park vegetation are pine forests with *Pinussylvestris* L. (Fig. 1) on vast fluvioglacial and alluvial sandy areas, large bogs (either preserved or drained for peat mining), marshes and alder forests (*Alnusglutinosa* (L.) Gaertn.) along slow rivers and areas deforested due to frequent fires. The territory of the Park is included in the List of Ramsar Wetlands of international importance by the decree of the Government of the Russian Federation No. 1050 dated 13.09.1994 (Fig. 2).

### Funding

The Meshchera National Park funded much of the fieldwork in 2017 and 2018. Part of the work was carried out within the framework of the State Budget Assignments (see "Acknowledgements" section), as well as in the framework of planned research of the Laboratory for the study and protection of biodiversity, Ryazan State University. The publication of the book (Shcherbakov et al. 2020) was carried out at the expense of the Meshchera National Park.

## Sampling methods

### Study extent

The Park (1,030 km^2^) was divided into 55 grid squares measuring 2.5' lat. × 5' long. or ca. 4.6 × 5.4 km following the scheme employed in adjacent Vladimir Oblast (Seregin 2014, Seregin 2021b). Consequent numbers from **01** to **55** were used to indicate the squares (Fig. 3). We performed 70 grid surveys, i.e. 43 surveys within 42 grid squares in 2017 and 27 surveys within 42 grid squares in 2018. In 2018, some surveys were fragmentary, since they were performed in spring or covered the aquatic flora only. As a result, we visited some grid squares repeatedly.

### Sampling description

We planned the routes according to the forest plans (1:25,000) kindly provided by the National Park’s Forest Department, as well as on the general topographic map of the region (1:200,000). The aim of each one-day route was to visit as many habitats as possible within a grid square.

We used standard printed forms with a list of the most widespread species for standard surveys of grid squares and for special surveys of aquatic plants. Some special surveys of lakes were undertaken using either a rubber boat or a motorboat. We collected herbarium vouchers for plants that could not be identified on site, as well as those of special floristic interest. Our collections are now preserved in the Herbaria of Moscow State University (MW) and Ryazan State University (RSU). The MW collections are fully available online (Seregin 2021c).

Data obtained by the authors in the field were imported into the distribution database on the Park's flora (MS Excel spreadsheet). The spreadsheet was checked against the final identifications of voucher specimens, if available. In 2020, it was used for the production of grid maps in "Flora of the Meschchersky National Park: checklist and atlas" (Shcherbakov et al. 2020). Examples of the published grid maps can be seen in Fig. 4.

The dataset combines two sources of initial records, namely, field records by the authors and earlier data from other sources (see "Temporal coverage" section). We completely revised the dataset in April-September 2021 in line with the call for data papers describing datasets from Russia by GBIF and finally published it (Shcherbakov et al. 2021).

The dataset includes only one occurrence per species per grid square; within a single grid square, more recent occurrences received priority. The numbers of species occurrences per grid square across all periods are given on the scheme (Fig. 5a). The second scheme shows our field efforts in the Park in 2017-2018 (Fig. 5b).

The data recording coverage was fairly even during our field work (Fig. 5b). The outstanding diversity recorded in three grid squares (424 species, 438 species and 408 species) seen on the first scheme (Fig. 5a) corresponds to the areas of intensive field studies made by earlier research missions in the 1950-1990s (see "Temporal coverage" section). A comparison of recent field data vs. unconfirmed historical records across the intensively studied areas (Table 1) gives an idea that one-day surveys may reveal roughly a half of all species present in each grid square with an obvious shift towards common and easy-to-observe species. Unconfirmed historical records add one to three quarters of species records in these grid squares with an average increase of ca. 157 species.

The higher plant diversity of the central part of the Meshchera Lowlands clearly reflects the level of human activities and the location of river valleys (Seregin 2013). These patterns could be better observed in the second scheme, based on our field data (Fig. 5b), since our grid surveys were performed uniformly as one-day trips. The long southern tip of the Park has neither roads nor residential areas, whereas its northern and central parts are quite highly populated with a line of diverse grid squares stretching from north to south along the Pra River. The low diversity of plants recorded on fringes of the Park reflects a collection bias due to a smaller proportion of studied areas falling within grid squares situated along the borders.

## Geographic coverage

### Description

The Meshchersky National Park, Ryazan Oblast, Russia

### Coordinates

54.5 and 55.5 Latitude; 39.5 and 41 Longitude.

## Taxonomic coverage

### Description

A dataset covers 817 taxa of vascular plants (tracheophytes), i.e. 806 species and hybrids, unidentified records of the genus *Alchemilla* L. and ten species aggregates which were not taxonomically resolved during field recording:

- *Pteridiumaquilinum* (L.) Kuhn agg. (represented by *Pteridiumpinetorum* C.N. Page & R.R. Mill),
- *Polygonumaviculare* L. agg. (several microspecies),
- *Chenopodiumalbum* L. agg. (several microspecies),
- *Ranunculusauricomus* L. agg. (several microspecies),
- *Plantagomajor* L. agg. (*Plantagomajor* L. s. str. and *Plantagouliginosa* F.W. Schmidt),
- *Plantagomedia* L. agg. (*Plantagomedia* L. s. str. and *Plantagourvillei* Opiz),
- *Sonchusarvensis* L. agg. (glandular *Sonchusarvensis* L. s. str. and glabrous *Sonchusuliginosus* M. Bieb.),
- *Tragopogondubius* Scop. agg. (most probably, *Tragopogondubius* Scop. s. str.),
- *Tragopogonpratensis* L. agg. (*Tragopogonpratensis* L. s. str. and *Tragopogonorientalis* L.),
- *Taraxacumofficinale* Wigg. agg. (several microspecies).

The nomenclature of the original source (Shcherbakov et al. 2020) follows the nomenclature in Seregin (2013). After automatic cross-linking of the original names with the GBIF backbone (GBIF Secretariat 2021), occurrences from the dataset represent 808 accepted species from 403 genera of 96 families.

### Taxa included

**Table:** 

Rank	Scientific Name	
phylum	Tracheophyta	

## Temporal coverage

**Data range:** 1868-1-01 – 2019-12-31.

### Notes

11,931 records out of 14,476 (i.e. 82.4%) were made during on-purpose field research in 2017 and 2018 by the authors (Fig. 5b).

**Figure 6.**
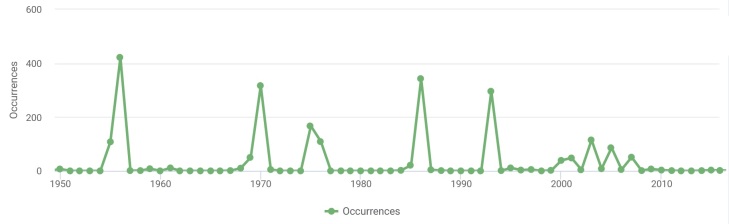
Temporal distribution of earlier grid records (1950-2016) unconfirmed by our field studies in 2017-2018. Peaks correspond to research missions of 1956, 1970, 1975, 1986 and 1993 (source: Shcherbakov et al. 2021).

There are also a number of earlier grid records in the dataset which were not confirmed during our field studies. For instance, 2,278 grid records (15.7%) were made in 1950-2016 with pronounced peaks in 1956, 1970, 1975, 1986 and 1993 (Fig. 6). Other records (267 occurrences or 1.8%) were either mobilised from scattered sources of 1868-1949 or left undated.

In 1955 and 1956, Klepikovsky District of Ryazan Oblast was intensively studied by the Ryazan Soil and Vegetation Expedition launched by the Moscow State University and headed by N.A. Prozorovsky and V.S. Govorukhin. We mobilised their 10 unpublished floristic surveys from the archive of N.B. Oktyabreva and five Master's Theses by the students of Geobotany Department, Moscow State University (1956).

In 1970 and 1975, the National Park was visited by research teams of the Meshchera Expedition of the Moscow University Botanic Garden, headed by V.N. Tikhomirov. We mobilised 14 unpublished floristic surveys performed by this research team (archive of N.B. Oktyabreva) and ca. 200 herbarium specimens preserved in the Moscow University Herbarium (MW) (Seregin 2021c). Large collections of this expedition were published in the form of point maps in the checklist for Ryazan Meshchera (Vodolazskaya et al. 1975). Later on, this research group published a two-volume guide for vascular plants of the Meshchera Lowlands (Alexeev et al. 1986, Kiseleva et al. 1987).

The Master's Thesis by T.O. Yanitskaya, a student of Higher Plants Department, Moscow State University, performed in 1986, included 360 vascular plants from the vicinity of Lake Negar. Peaks of 1993 and some additional dents of 2000-2007 correspond to earlier research missions by A.V. Shcherbakov and M.V. Kazakova, the authors of this paper.

## Usage licence

### Usage licence

Creative Commons Public Domain Waiver (CC-Zero)

### IP rights notes

This work is licensed under a Creative Commons Attribution (CC-BY) 4.0 License.

## Data resources

### Data package title

A grid database on vascular plant distribution in the Meshchersky National Park, Ryazan Oblast, Russia

### Resource link

https://doi.org/10.15468/mnv9rt

### Alternative identifiers

10.15468/mnv9rt, c8916e4d-cfa0-41e1-81d1-fa35f49d5b1e, https://depo.msu.ru/ipt/resource?r=ryazan

### Number of data sets

1

### Data set 1.

#### Data set name

A grid database on vascular plant distribution in the Meshchersky National Park, Ryazan Oblast, Russia

#### Data format

Darwin Core

#### Number of columns

44

#### Download URL

https://depo.msu.ru/ipt/archive.do?r=ryazan

#### Description

A dataset contains 14,476 records of 817 taxa of vascular plants from the Meshchersky National Park (Ryazan Oblast, Russia) mostly made by A.V. Shcherbakov et al. in 2017-2018. The dataset is based on a grid scheme with 55 squares (2.5' lat. × 5' long., WGS84 or ca. 25 km^2^). In 2020, the dataset was used to produce maps in the printed atlas (Shcherbakov et al. 2020).

**Data set 1.** 

Column label	Column description
occurrenceID	An identifier for the occurrence. A variable constructed from a combination of two identifiers (datasetID and catalogNumber). For example, "urn:lsid:biocol.org:col:15550:08:00001".
dcterms:type	The nature or genre of the resource. A constant ("Dataset").
dcterms:modified	The most recent date-time on which the resource was changed. A constant ("2021-04-16").
dcterms:language	A language of the resource. A constant ("en" = English).
dcterms:license	A legal document giving official permission to do something with the resource. A constant ("http://creativecommons.org/licenses/by/4.0/legalcode").
dcterms:rightsHolder	A person or organisation owning or managing rights over the resource. A constant ("Moscow State University").
dcterms:accessRights	Information about who can access the resource or an indication of its security status. A constant ("Use under CC BY 4.0").
institutionID	An identifier for the institution having custody of the object(s) or information referred to in the record. A constant ("http://grbio.org/institution/moscow-state-university" for the Moscow State University).
collectionID	An identifier for the collection or dataset from which the record was derived. A constant ("urn:lsid:biocol.org:col:15550" for the Moscow University Herbarium).
datasetID	An identifier for the set of data. May be a global unique identifier or an identifier specific to a collection or institution. A constant ("urn:lsid:biocol.org:col:15550:08").
institutionCode	The name (or acronym) in use by the institution having custody of the object(s) or information referred to in the record. A constant ("Moscow State University").
collectionCode	The name, acronym, coden or initialism identifying the collection or dataset from which the record was derived. A constant ("MW" for the Moscow University Herbarium).
datasetName	The name identifying the dataset from which the record was derived. A constant ("A grid database on vascular plant distribution in the Meshchersky National Park, Ryazan Oblast, Russia").
ownerInstitutionCode	The name (or acronym) in use by the institution having ownership of the object(s) or information referred to in the record. A constant ("Moscow State University").
basisOfRecord	The specific nature of the data record - a subtype of the dcterms:type. A constant ("Human Observation")
informationWithheld	Additional information that exists, but that has not been shared in the given record. A constant ("Occurrence is placed in the grid square centroid; real coordinates, event date, habitat details and voucher information (if present) are obscured.").
dataGeneralizations	Actions taken to make the shared data less specific or complete than in its original form. A constant ("Occurrence is placed in the grid square (2.5′ lat. x 5.0′ long.) centroid. Only one record per grid per taxon is included into the dataset (i.e. the latest one). A year is given instead of a real event date.").
catalogNumber	An identifier (preferably unique) for the record within the dataset or collection. A variable (for example, "000001").
recordedBy	A list (concatenated and separated) of names of people, groups or organisations responsible for recording the original occurrence. A variable (for example, "A.V. Shcherbakov | et al.").
occurrenceStatus	A statement about the presence or absence of a taxon at a location. A constant ("present").
associatedReferences	A list (concatenated and separated) of identifiers (publication, bibliographic reference, global unique identifier, URI) of literature associated with the Occurrence. A constant ("Shcherbakov A.V., Kazakova M.V., Lyubeznova N.V., Pastushenko A.D. 2020. Flora natsionalnogo parka "Meshcherskii": konspekt i atlas. Moscow: Galleya-Print. 285 p. (http://herba.msu.ru/shipunov/school/books/scherbakov2020_fl_nats_parka_mescherskij.djvu)").
eventDate	The date or interval during which an event occurred. For occurrences, this is the date when the event was recorded. A year is indicated in this dataset instead of a real date. A variable (for example, "2017").
eventRemarks	Comments or notes about the event. A variable (three options: "Standard survey period 1860-1949", "Standard survey period 1950-1999", "Standard survey period 2000-2019").
higherGeography	A list (concatenated and separated) of geographic names less specific than the information captured in the locality term. A constant ("Europe | Russian Federation | Ryazan Oblast").
continent	The name of the continent in which the location occurs. A constant ("Europe").
country	The name of the country or major administrative unit in which the location occurs. A constant ("Russian Federation").
countryCode	The standard code for the country in which the location occurs. A constant ("RU").
stateProvince	The name of the next smaller administrative region than country (state, province, canton, department, region etc.) in which the location occurs. A constant ("Ryazan Oblast").
verbatimLocality	The original textual description of the place. A variable with grid square index. For example, "Grid square 05".
locationAccordingTo	Information about the source of this location information. Could be a publication (gazetteer), institution or team of individuals. A constant ("Shcherbakov A.V., Kazakova M.V., Lyubeznova N.V., Pastushenko A.D. 2020. Flora natsionalnogo parka "Meshcherskii": konspekt i atlas. Moscow: Galleya-Print. 285 p. (http://herba.msu.ru/shipunov/school/books/scherbakov2020_fl_nats_parka_mescherskij.djvu)").
decimalLatitude	The geographic latitude (in decimal degrees, using the spatial reference system given in geodeticDatum) of the geographic centre of a location. A variable (latitude of a grid square centroid). For example, "55.27083".
decimalLongitude	The geographic longitude (in decimal degrees, using the spatial reference system given in geodeticDatum) of the geographic centre of a location. A variable (longitude of a grid square centroid). For example, "40.29167".
geodeticDatum	The ellipsoid, geodetic datum or spatial reference system (SRS) upon which the geographic coordinates given in decimalLatitude and decimalLongitude are based. A constant ("WGS84").
coordinateUncertaintyInMeters	The horizontal distance (in metres) from the given decimalLatitude and decimalLongitude describing the smallest circle containing the whole of the location. A constant ("3500" or an average distance between a grid square centroid and a grid square corner).
georeferencedBy	A list (concatenated and separated) of names of people, groups or organisations who determined the georeference (spatial representation) of the location. A constant ("A.V. Shcherbakov").
identifiedBy	A list (concatenated and separated) of names of people, groups or organisations who assigned the Taxon to the subject. A variable (for example, "A.V. Shcherbakov | et al.").
scientificName	The full scientific name, with authorship and date information, if known. A variable (for example, "*Matteucciastruthiopteris* (L.) Tod.").
kingdom	The full scientific name of the kingdom in which the taxon is classified. A constant ("Plantae").
phylum	The full scientific name of the phylum or division in which the taxon is classified. A constant ("Tracheophyta").
family	The full scientific name of the family in which the taxon is classified. A variable (for example, "Lamiaceae").
genus	The full scientific name of the genus in which the taxon is classified. A variable (for example, "*Matteuccia*").
taxonRank	The taxonomic rank of the most specific name in the scientificName. A variable (three options: "Species", "Genus", "speciesAggregate").
nomenclaturalCode	The nomenclatural code (or codes in the case of an ambiregnal name) under which the scientificName is constructed. A constant ("International Code of Nomenclature for algae, fungi and plants").
taxonomicStatus	The status of the use of the scientificName as a label for a taxon. A constant ("accepted").

## Figures and Tables

**Figure 1a.**
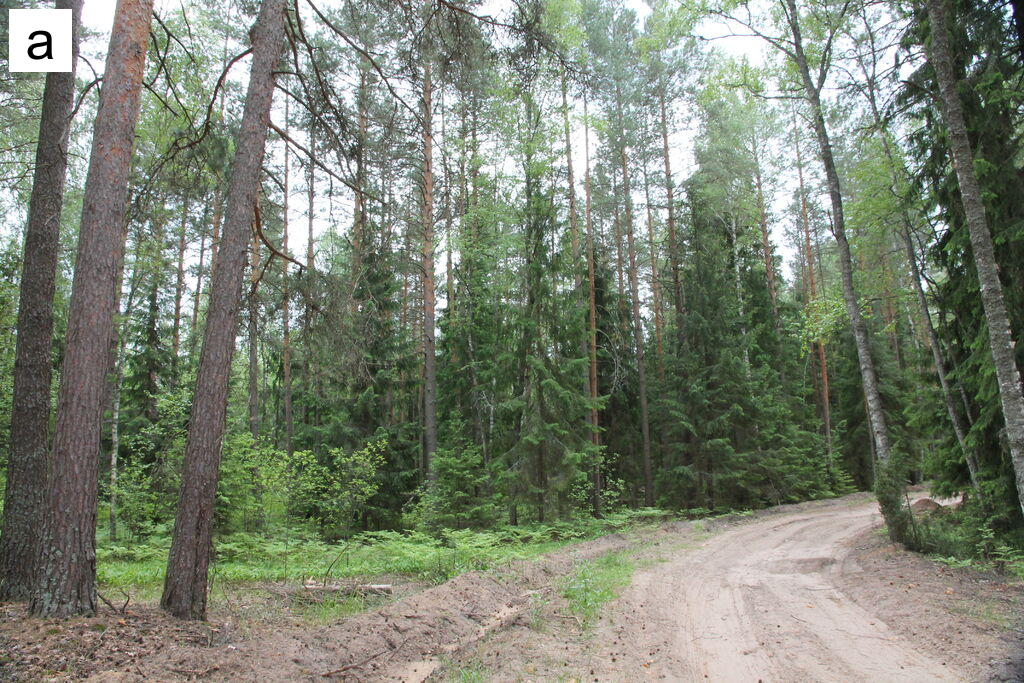
Pine forest with spruce (*Piceaabies* (L.) H.Karst.) undergrowth near Zavodskaya Sloboda. Photo by M.V. Kazakova.

**Figure 1b.**
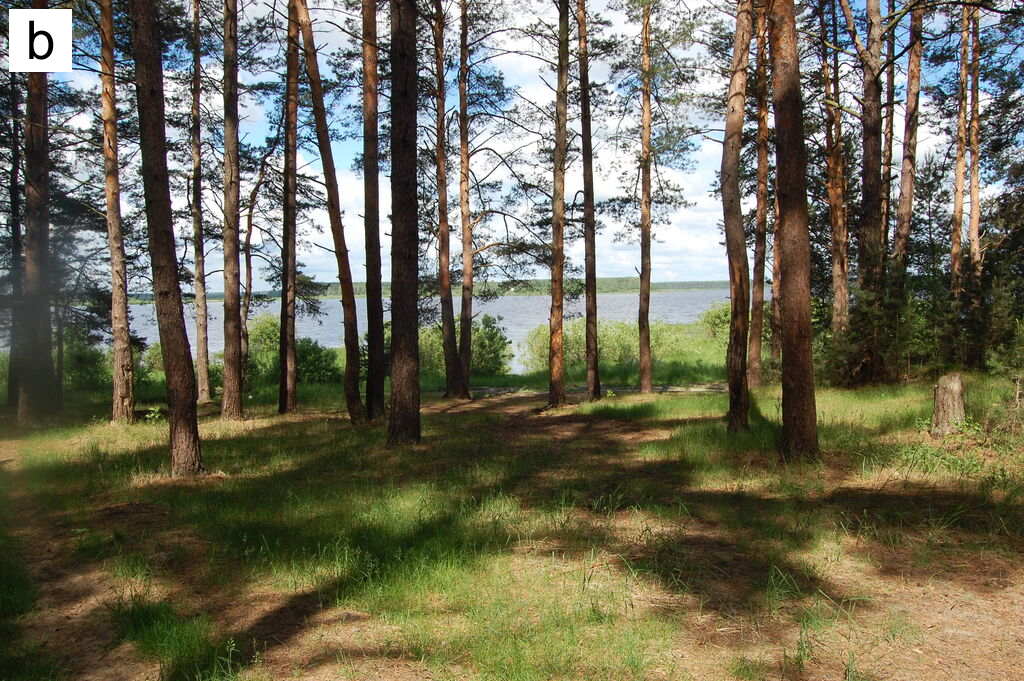
Sparse pine forest dominated by grasses (a camping site) near Lake Sokorevo. Photo by M.V. Kazakova.

**Figure 2a.**
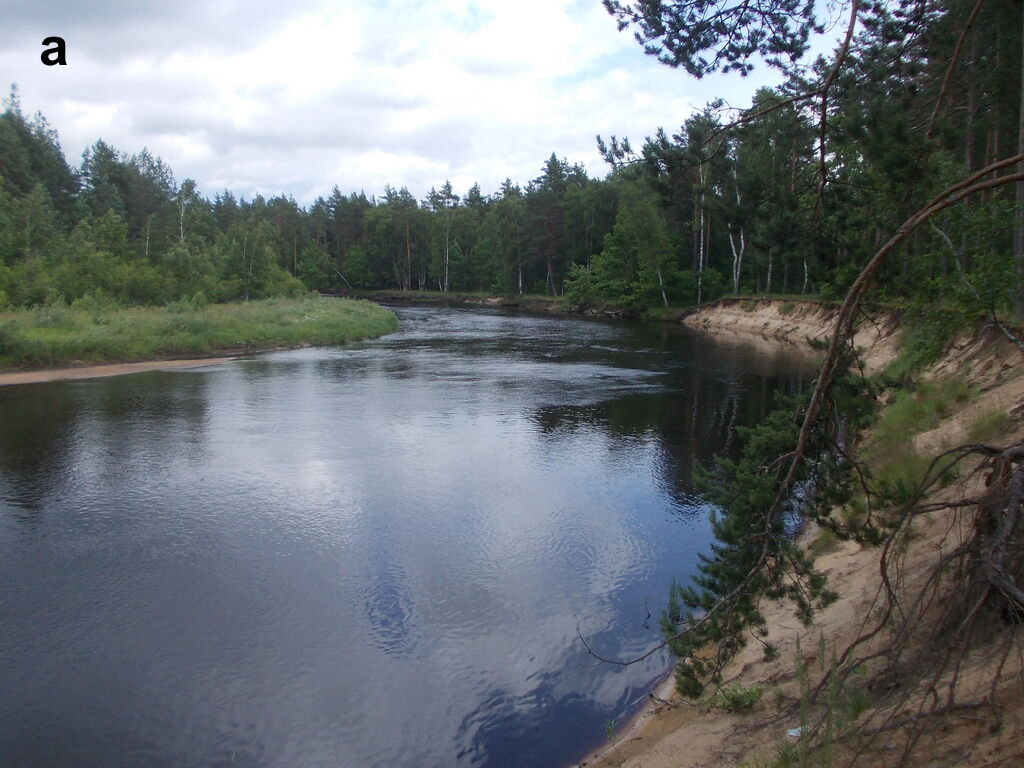
The Pra River with pine forests on sandy ground. Photo by N.V. Lyubeznova.

**Figure 2b.**
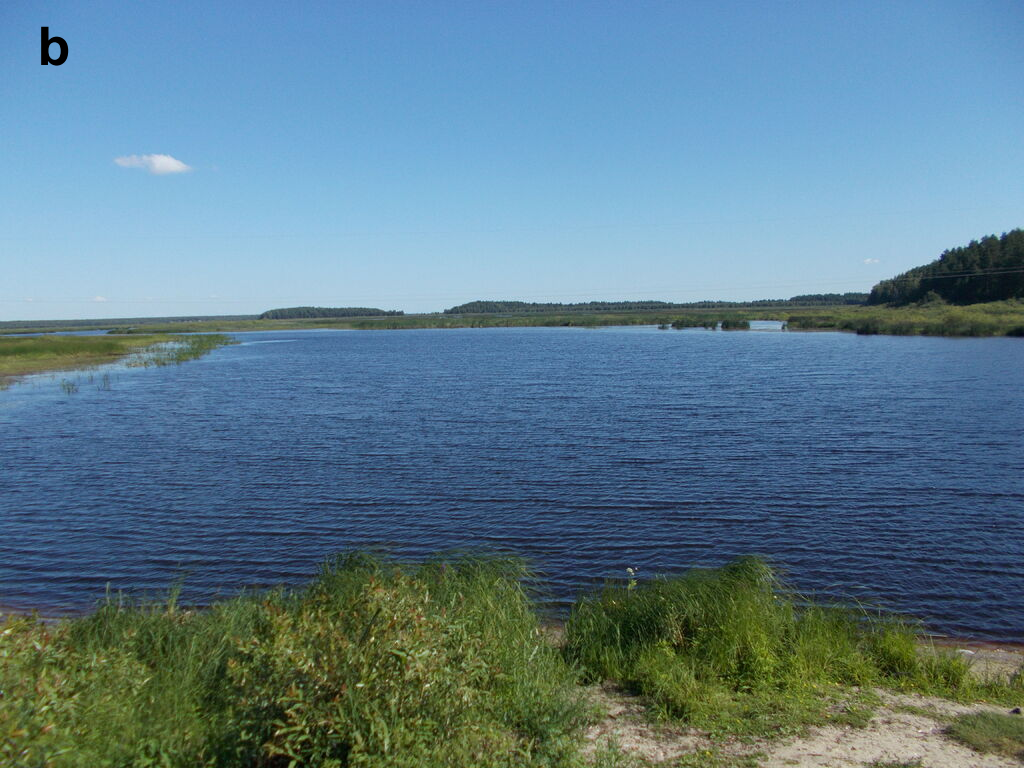
Lake Velikoye. Photo by N.V. Lyubeznova.

**Figure 3a.**
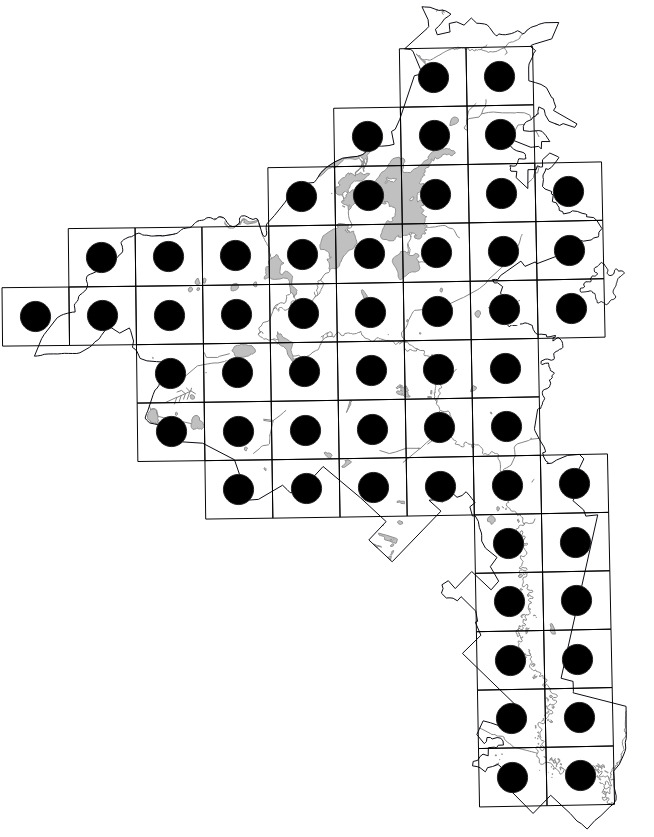
Positions of the 55 centroids.

**Figure 3b.**
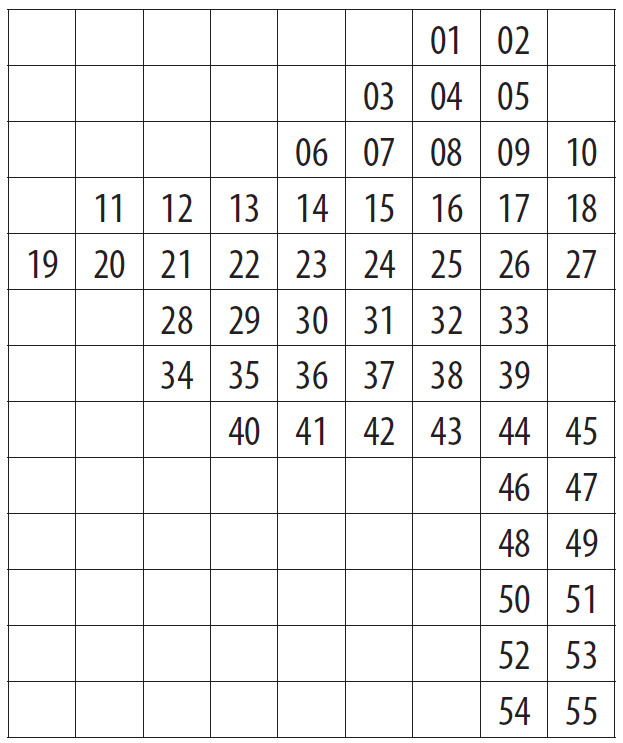
Indexes of the grid squares.

**Figure 4a.**
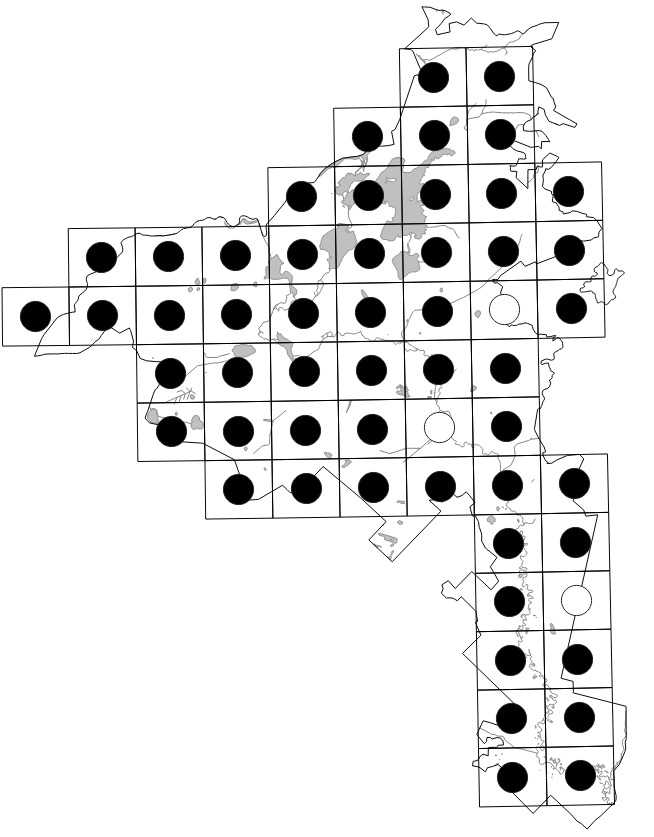
*Dryopteriscarthusiana* (Vill.) H.P. Fuchs, a widespread native plant with historic records in three grid squares.

**Figure 4b.**
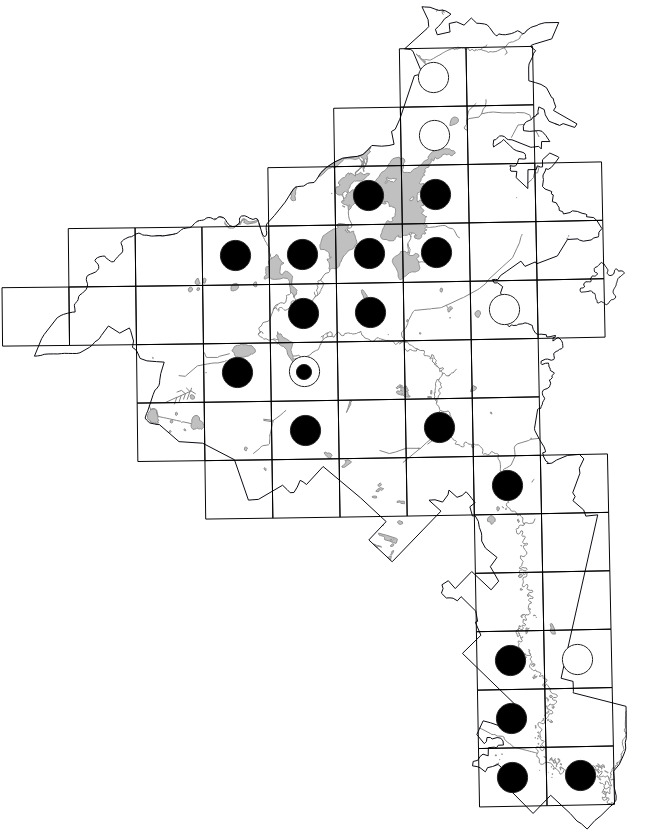
*Rorippaamphibia* (L.) Besser, a typical native species of the river valleys.

**Figure 4c.**
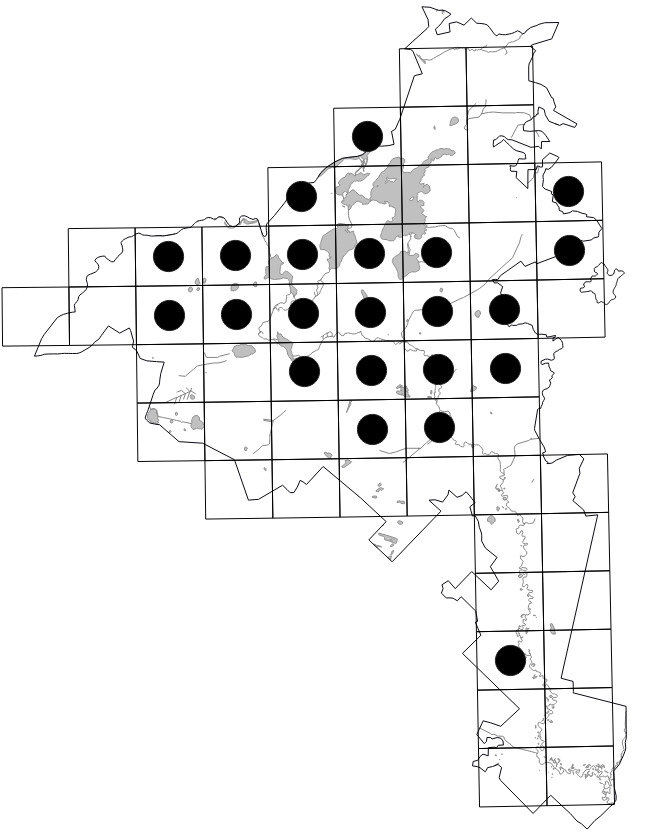
*Amelanchierspicata* (Lam.) K. Koch, an invasive alien of pine forests.

**Figure 4d.**
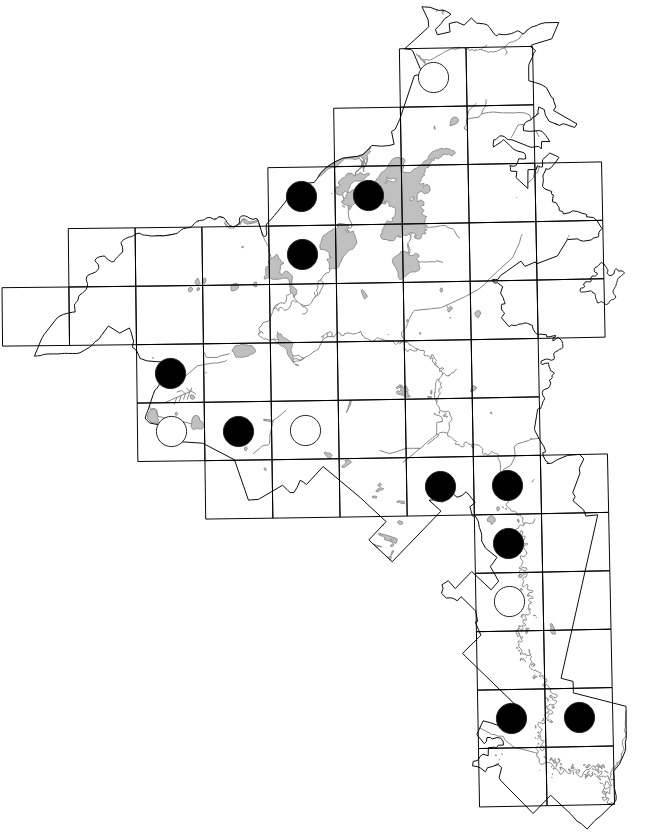
*Pulsatillapatens* (L.) Mill., a legally-protected species typical of pine forests on ancient alluvial plains.

**Figure 5a.**
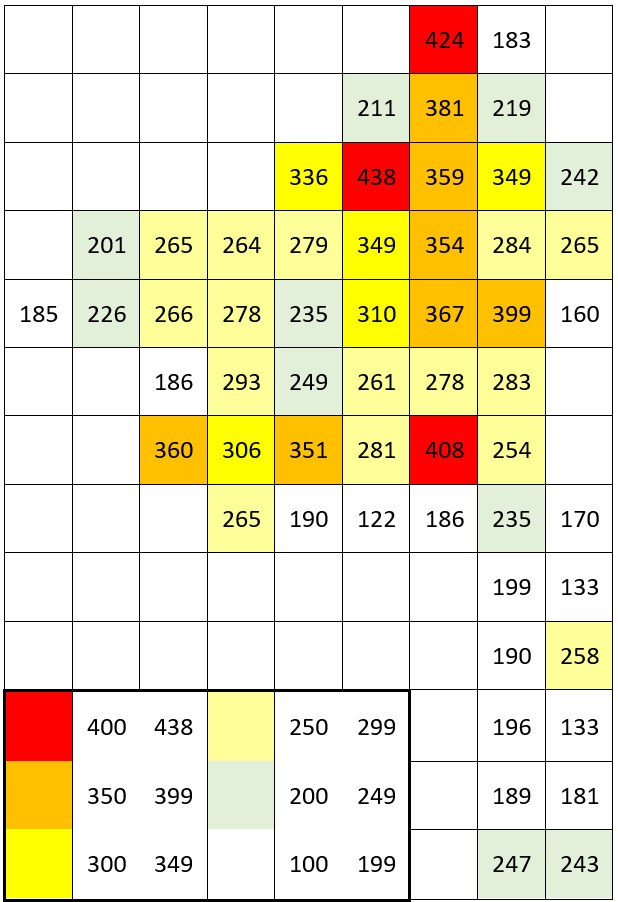
All data (1869-2019).

**Figure 5b.**
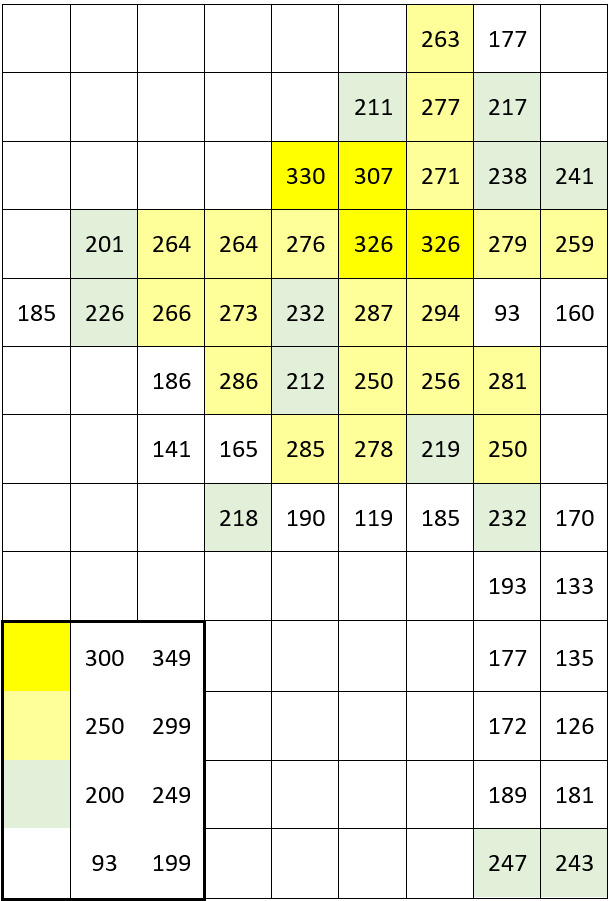
Recent field data by the authors (2017-2018).

**Table 1.** Insufficiently studied grid squares with a lower proportion of recent records.

**Grid square**	**Our field records (2017-2018)**	**All records** **(1869-2019)**	**Proportion of our field records**, %
Grid square 26	93	399	23
Grid square 34	141	360	39
Grid square 49	135	258	52
Grid square 35	165	306	54
Grid square 38	219	408	54
Grid square 01	263	424	62
Grid square 09	238	349	68
Grid square 07	307	438	70
Grid square 04	277	381	73
Grid square 08	271	359	75
